# Effects of *Hahella chejuensis*-Derived Prodigiosin on UV-Induced ROS Production, Inflammation and Cytotoxicity in HaCaT Human Skin Keratinocytes

**DOI:** 10.4014/jmb.2011.11024

**Published:** 2020-12-23

**Authors:** Jieun Lee, Hyun Ju Kim, Sang Jun Lee, Moo-Seung Lee

**Affiliations:** 1 Environmental Diseases Research Center, Korea Research Institute of Bioscience and Biotechnology (KRIBB), Daejeon 34141, Republic of Korea; 2 Department of Systems Biotechnology, Chung-Ang University, Anseong 17546, Republic of Korea; 3 Department of Biomolecular Science, KRIBB School of Bioscience, Korea University of Science and Technology (UST), Daejeon 34113, Republic of Korea

**Keywords:** Prodigiosin, *Hahella chejuensis*, kratinocyte, UV irradiation

## Abstract

Prodigiosins, which are natural tripyrrole red pigments and synthetic derivatives, reportedly have multiple biological effects mainly on various types of cancer cells. However, the effects of bacterial prodigiosin on non-cancerous HaCaT human skin keratinocytes have not been reported. Therefore, the present study aimed to investigate the functional activities of prodigiosin derived from cultures of the bacterium *Hahella chejuensis* in HaCaT cells. Cell viability, the cell proliferation rate, and reactive oxygen species (ROS) production in vitro were assayed following treatment of HaCaT cells with prodigiosin. Prodigiosin did not cause cytotoxicity and notably increased proliferation of HaCaT cells. Furthermore, prodigiosin reduced ultraviolet (UV) irradiation-induced ROS production and the inflammatory response in HaCaT cells. More importantly, prodigiosin reduced matrix metalloproteinase-9 expression and increased collagen synthesis in UV-irradiated HaCaT cells, demonstrating that it elicits anti-aging effects. In conclusion, our results reveal that *H. chejuensis*-derived prodigiosin is a potential natural product to develop functional cosmetic ingredients.

## Introduction

Microorganisms produce metabolic products that have various activities [1]. These products are called microbial secondary metabolites and mainly promote cell growth, inhibit enzymatic activities, and modulate the immune response [1, 2]. Most of these metabolites are natural bioactive products generated by bacteria [3]. Prodigiosin is a microbial secondary metabolite and red pigment mainly produced by *Serratia* and *Vibrio* species and a few Gram-positive bacteria specifically in the later stages of bacterial growth [4]. The prodigiosin is clustered by the biosynthesis of prodiginines via a bifurcated pathway building up in the enzymatic condensation. Among prodigiosin-producing bacteria, *Hahella chejuensis* is a special marine bacterium collected from marine sediment [5]. Although prodigiosin derived from *H. chejuensis* has been poorly investigated due to difficult culture growth condition with diverse environmental factors and complicated extract conditions, *H. chejuensis* produces large amounts of prodigiosin as beneficial natural products in the ocean.

Human skin is a major organ and functions as a barrier to protect the body against diverse environmental factors [6]. The skin is considered to be a major component of the immune system because it protects the host by providing a physical barrier and there is a complex network comprising immune and non-immune cells and skin structures. Consequently, skin injuries are rapidly repaired. Despite this defensive function of the skin, ultraviolet (UV) radiation damages living organisms by increasing the level of intracellular reactive oxygen species (ROS) [7]. Exposure to UV radiation is a major cause of various human skin injuries and diseases such as inflammation, aging, and cancer [8]. UV radiation damages skin keratinocytes and therefore the skin must be protected against such radiation to prevent skin injuries and diseases.

Suryawanshi *et al*. [9] reported that prodigiosin is one of the pigments produced by microorganisms found in habitats exposed to a high level of UV radiation, as well as being produced in the dark, and it protects against such radiation. This indicates that prodigiosin can be used to develop natural cosmetic materials that protect the skin against UV radiation. Lin *et al*. [10] reported that natural and organic resources and materials are being increasingly used in the cosmetic industry. However, it is difficult to identify natural materials whose supply is unlimited. Prodigiosin derived from *H. chejuensis*, a marine microorganism found in habitats exposed to a high level of UV radiation, may be a natural compound with an unlimited supply that can prevent UV-induced skin damage. In addition, *H. chejuensis*-derived prodigiosin is suitable for industrial biotechnology applications because it is easy, fast, and inexpensive to cultivate microorganisms and harvest prodigiosin.

This study aimed to assess the effects of *H. chejuensis*-derived prodigiosin on the ROS level, pro-inflammation, and cytotoxicity in HaCaT human skin keratinocytes. These findings may enhance our understanding of the potential application of prodigiosin as an active natural product derived from marine bacteria.

## Materials and Methods

### Preparation of Prodigiosin from *H. chejuensis*

*H. chejuensis* (KCTC 2396) cells were streaked and grown on Marine Agar (Cat. No. 2216; BD Difco, USA) at 30°C. A single colony of *H. chejuensis* was inoculated into 25 ml Marine Broth and cultured at 30°C for 48 h with shaking at 200 rpm. Starter cultures (1%) were transferred to 1 L flasks filled with 100 ml Marine Broth and red polyurethane (PU) foam cubes (Jeongan Sponge Co., Ltd., Korea; density, 25 kg/m^3^; size, ~1 cm^3^) to extract prodigiosin. Cultures were incubated at 30°C for 48 h with shaking at 200 rpm, and then PU foam cubes were filtered through gauze to remove the culture medium. Prodigiosin was extracted from PU foam cubes using a Soxhlet extractor and ethanol. The concentration of prodigiosin was measured by high-performance liquid chromatography (Agilent 1100 Series HPLC System; Agilent, USA) using a C18 column (WAT05427, 100 Å, 5 μm, 4.6 × 250 mm; Waters Corp., USA). Isocratic elution was performed at 25°C with a flow rate of 0.8 ml/min using a methanol:acetonitrile:distilled water (1:1:2, v/v) solution (pH adjusted to 3.6 using acetic acid) as the mobile phase. The eluted prodigiosin was concentrated, lyophilized, and pulverized and its final purity was 93.2%.

### Cell Culture

The HaCaT human skin keratinocyte cell line was kindly gifted by the Korea Research Institute of Bioscience and Biotechnology (KRIBB, Republic of Korea) and cultured in 5% CO_2_ at 37°C in complete Dulbecco’s modified Eagle’s medium (DMEM; Corning, USA) containing 100 U/ml penicillin, 100 μg/ml streptomycin, 10% heat-inactivated fetal bovine serum (FBS; GE Healthcare Life Sciences, Australia), and CaCl_2_ (final Ca^2+^ concentration of 0.33 mM) [11].

### Measurement of Cytotoxicity and Cell Viability

HaCaT cells were irradiated with UV (40 mJ/cm^2^) in PBS and treated with prodigiosin (50 or 100 ng/ml) in DMEM and then 50 μl culture medium was mixed with 50 μl reaction mixture provided with a CyQUANT LDH Cytotoxicity Assay Kit (Thermo Fisher Scientiﬁc, USA) at room temperature for 30 min in the dark. Thereafter, 50 μl stop solution was added by gentle pipetting. Absorbance at 490 and 680 nm was measured.

A Quanti-Max WST-8 Cell Viability Assay Kit (Biomax Ltd., Korea) was used to analyze cell viability according to the manufacturer’s instructions. HaCaT cells (4 × 10^4^/well) were seeded into 96-well plates. All assays were performed in triplicate. Cells in each well were suspended in 100 μl fresh medium containing various concentrations of prodigiosin and then were reseeded. After UV irradiation or prodigiosin treatment as described above, 10 μl WST-8 was added to each well, samples were incubated for 2 h, and absorbance at 450 nm was measured.

### Flow Cytometric Analysis of the Cell Cycle

As described in [11], HaCaT cells (3 × 10^5^/well) were seeded into 12-well plates and irradiated with UV (40 mJ/cm^2^) in PBS and treated with prodigiosin (50 ng/ml) in DMEM. Cells were harvested after incubation with 10× Trypsin-EDTA (0.5%, no phenol red; Thermo Fisher Scientiﬁc) diluted in PBS for 5 min at 37°C and resuspended in an equal volume of fresh DMEM containing 10% FBS. Samples were centrifuged at 300 ×*g* for 5min and washed with PBS. Cell pellets were fixed in cold 70% ethanol and kept at −20°C for 7 days until cell cycle analysis. Ethanol-fixed cells were washed twice with 250 μl PBS. Cell pellets were resuspended in 200 μl Muse Cell Cycle Reagent and incubated at room temperature for 30 min in the dark. The cell suspension was transferred to a 1.5 ml microcentrifuge tube without a cap. The cell cycle was analyzed using a Muse Cell Analyzer (Millipore Corporation, USA).

### Reverse Transcription-quantitative PCR (RT-qPCR)

Each 20 μl RT-qPCR sample contained 2−4 ng/μl RNA template, 12.5 μl of a commercially available master mix (RealHelix qRT-PCR Kit; Nanohelix Co., Republic of Korea), and 100 pmol/ml of each primer. The primer sequences are provided in Table 1. ACTIN was included as an internal control. The real-time PCR cycling conditions included a common amplification step with an initial cycle for cDNA synthesis at 50°C for 3 min and initial denaturation at 95°C for 15 min, followed by 42 cycles of denaturation at 95°C for 20 sec and annealing and extension at 60°C for 60 sec. SYBR Green was used to quantify the results. Data were analyzed using LightCycler 96 System Software 1.1 (Roche Diagnostics GmbH, Germany), and relative expression levels were normalized against ACTIN as internal control.

### Measurement of ROS

ROS were detected by fluorescence microscopy using CM-H_2_DCFDA (Thermo Fisher Scientific). HaCaT cells were cultured in 4-well chambered cell culture slides in DMEM growth medium containing antibiotics and 10%FBS. After UV irradiation (40 mJ/cm^2^) in PBS, cells were stained with Hoechst 33342 (Thermo Fisher Scientific) at 37°C for 5 min. Cells were treated with prodigiosin (50, 100, and 200 ng/ml) in DMEM for 4 h and then incubated in warm PBS containing 5 μM CM-H_2_DCFDA at 37°C for 25 min. Cells were washed twice with PBS and fixed with 4% paraformaldehyde at 37°C for 10 min. The fluorescence intensity of CM-H_2_DCFD was measured using an EVOS M5000 fluorescence microscope at 40× magnification and quantified using on-board software. The fluorescence intensity was compared with that in UV-irradiated control cells (set to 100%).

### Enzyme-Linked Immunosorbent Assays (ELISAs)

The level of pro-collagen type I in cell culture media was quantified using a Human Pro-Collagen I alpha 1 ELISA Kit (Fluorescent) according to the manufacturer’s instructions (Abcam plc., UK). The fluorescence intensity at 535/590 nm (excitation/emission) was measured using a fluorescence plate reader (Tecan Infinite F200 PRO Multimode Reader; Tecan, Switzerland).

Levels of tumor necrosis factor (TNF)-α, interleukin (IL)-6, IL-8, and CCL2 in cell culture media were quantified using Human ELISA Kits (Thermo Fisher Scientific) according to the manufacturer’s instructions. Absorbance at 450 nm was measured using a spectrophotometer (SpectraMax M5e Microplate Reader; Molecular Devices, USA).

### Statistical Analysis

Data were analyzed using GraphPad Prism software and reported as mean ± standard error of the mean (SEM). Statistical analyses were performed using the Student’s *t*-test (for paired or unpaired samples as appropriate). A *p*-value < 0.05 was considered significant.

## Results and Discussion

### Prodigiosin Increases Proliferation of Keratinocytes

**Fig. 1:**
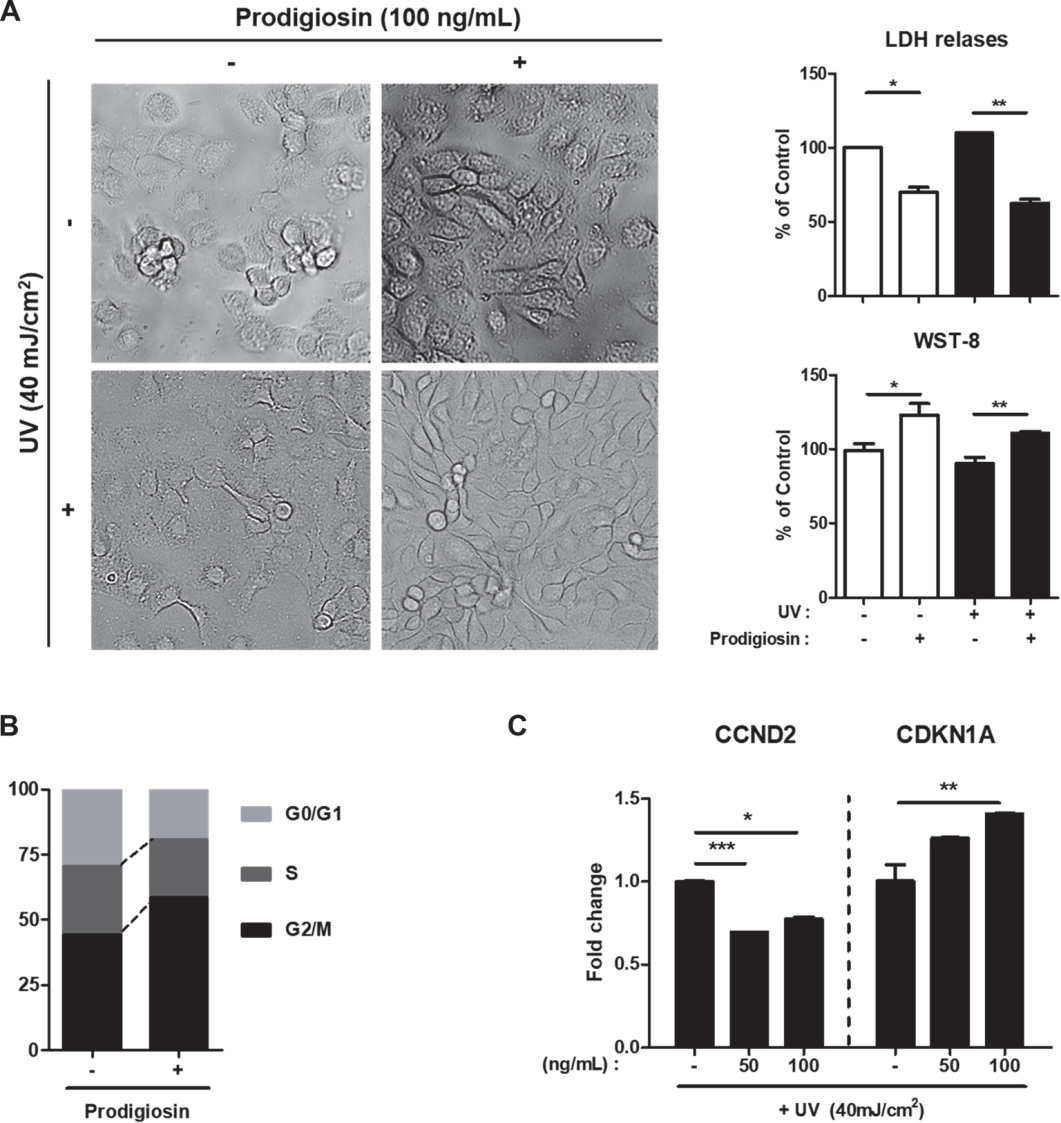
Prodigiosin alters the morphology and cell cycle of UV-irradiated HaCaT cells. (**A**) HaCaT cells maintained in low CaCl_2_ medium or irradiated with 40 mJ/cm^2^ UV light were incubated in medium containing 0.2% DMSO or 100 ng/ml prodigiosin for 1 h. (Left panel) Effect of prodigiosin on cellular morphology. (Right upper panel) The cellular protective effect and (right lower panel) cytotoxicity of prodigiosin were evaluated using the CyQUANT LDH Cytotoxicity Assay and a Quanti-MAX WST-8 Cell Viability Assay Kit. Data represent mean ± SEM of three replicates. Statistical analyses were performed using the two-tailed Student’s *t*-test. *p < 0.05 and **p < 0.001. (**B**) HaCaT cells were irradiated with 40 mJ/cm^2^ UV light and treated with 50 ng/ml prodigiosin for 1 h. The cell cycle was investigated using a Muse Cell Cycle Kit, and samples were analyzed using Muse 1.5 Analysis software. (**C**) mRNA levels of cell cycle-related genes (CCND2 and CDKN1A) in HaCaT cells irradiated with 40 mJ/cm^2^ UV light and treated with 50 or 100 ng/ml prodigiosin were examined by RT-qPCR. ACTIN was used as an internal control. Data represent mean ± SEM of two independent experiments. Statistical analyses were performed using the two-tailed Student’s *t*-test. *p < 0.05, **p < 0.001, and ***p < 0.0001.

Previous studies reported that prodigiosin derived from the *Serratia* genus has a few activities [12], whereas that derived from the marine bacterium *H. chejuensis* does not [13]. HaCaT human keratinocytes were treated with various concentrations (0−200 ng/ml) of *H. chejuensis*-derived prodigiosin dissolved in DMSO for 1 or 4 h and irradiated with UV (40mJ/cm^2^) [14] to develop an in vitro model of UV radiation-induced skin injury. We studied the effects of prodigiosin on cellular morphology, cytotoxicity, and cell proliferation. Following treatment with 100 ng/ml prodigiosin for 1 h, both UV-irradiated and non-irradiated cells migrated more and aggregated to form colonies (Fig. 1A, left panel). Treatment with 100 ng/ml prodigiosin for 1 h significantly decreased cytotoxicity and increased cell proliferation (Fig. 1A, right panel). In summary, prodigiosin reduced cytotoxicity, protected against UV irradiation, and enhanced proliferation of keratinocytes.

We next examined the impact of prodigiosin on the cell cycle of HaCaT cells. Upon UV irradiation, the percentages of cells in G0/G1 and S phases were significantly lower among prodigiosin-treated cells than among non-treated cells (Fig. 1B). We performed RT-qPCR to analyze expression of two cell cycle-related genes [15] in UV-irradiated HaCaT cells treated with 50 or 100 ng/ml prodigiosin. As expected, mRNA expression of cyclin D2 (CCND2), which is involved in the G1/S phase transition [16], was significantly lower in prodigiosin-treated cells than in non-treated cells (Fig. 1C). mRNA expression of cyclin-dependent kinase N1A (CDKN1A), which inhibits progression from G1 phase to S phase, was 1.5-fold higher in prodigiosin-treated cells than in non-treated cells. G2/M phase directly precedes mitosis, a period of cell growth [17]. The percentage of cells in G2/M phase was 31.75% higher among prodigiosin-treated cells than among non-treated cells after UV irradiation (Fig. 1B). This indicates that prodigiosin prevents UV-induced injury of cells, which is consistent with the results concerning cellular morphology presented in Fig. 1A. Therefore, we conclude that prodigiosin facilitates cell cycle progression and enhances proliferation of HaCaT cells and thereby reduces cellular injury.

 Many previous studies reported that a prodigiosin-producing S. marcescens strain elicits anticancer effects by inducing apoptosis or causing cytotoxicity in cancer cells [18-20]. Our study is the first to report the mechanisms underlying the cellular activities of a prodigiosin-producing *H. chejuensis* strain. We showed that prodigiosin reduced cytotoxicity and increased proliferation of keratinocytes and thereby protected against UV irradiation.

### Prodigiosin Reduces the ROS Level in UV-Irradiated Keratinocytes

**Fig. 2:**
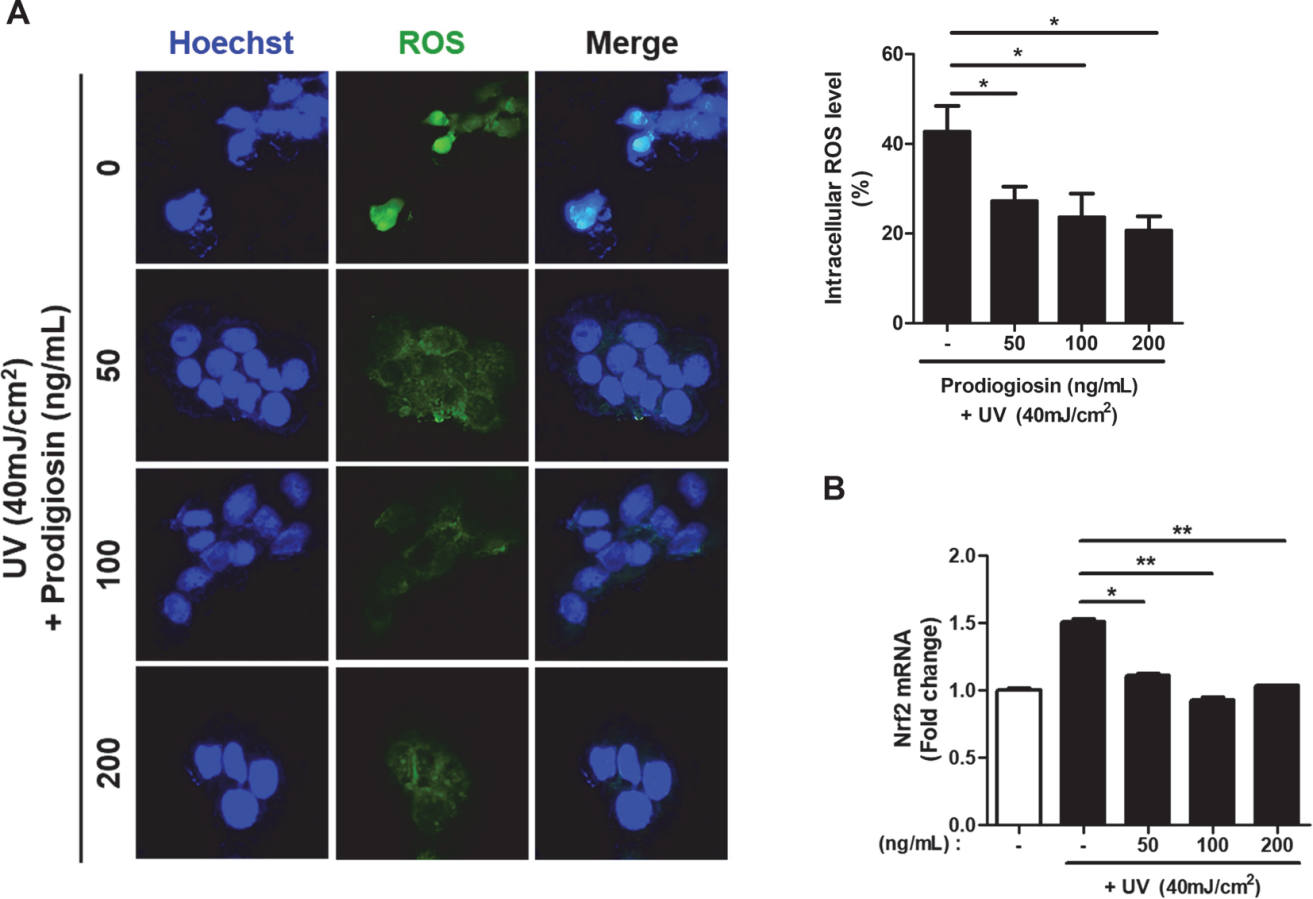
Prodigiosin reduces UV irradiation-induced ROS production in HaCaT cells. HaCaT cells were irradiated with 40 mJ/cm^2^ UV light and treated with 0−200 ng/ml prodigiosin for 4 h. (**A**, left panel) Representative images of CM-H_2_DCFDA staining and (**A**, right panel) quantitative analysis of the fluorescence intensity of CM-H_2_DCFDA in five randomly selected images acquired using an EVOS M5000 microscope. (**B**) mRNA levels of NRF2 determined by RT-qPCR in non-irradiated (white bar) and UV-irradiated (black bars) cells. Data represent mean ± SEM of two independent experiments. Statistical analyses were performed using the two-tailed Student’s *t*-test. *p < 0.05 and **p < 0.001.

To investigate whether prodigiosin reduces UV-induced ROS generation [21], we measured the fluorescence intensity of the ROS indicator CM-H_2_DCFDA using a fluorescence microscope. Treatment with prodigiosin (0–200 ng/ml) for 1 h significantly reduced the fluorescence intensity of CM-H_2_DCFDA in UV-irradiated (40 mJ/cm^2^) HaCaT cells in a dose-dependent manner (Fig. 2A). The fluorescence intensity of CM-H_2_DCFDA in UV-irradiated cells treated with 200 ng/ml prodigiosin was half of that in non-treated UV-irradiated cells. These results show that prodigiosin effectively scavenges UV radiation-induced ROS in HaCaT cells.

Furthermore, mRNA expression of NRF2 [22, 23] was quantified to evaluate the response to ROS-induced oxidative stress. mRNA expression of NRF2 was significantly lower in prodigiosin-treated cells than in non-treated cells following UV irradiation (Fig. 2B). These results indicate that prodigiosin enhances the cellular response to ROS-induced oxidative stress by regulating transcription of NRF2. Braun *et al*. [22] firstly reported that the transcription factor NRF2 plays an important role in wound healing by keratinocytes in the presence of ROS. Our results suggest that prodigiosin reduces the ROS level in UV-irradiated keratinocytes and that this effect is related to NRF2 expression.

### Prodigiosin Inhibits Photoaging in UV-Irradiated Keratinocytes

**Fig. 3:**
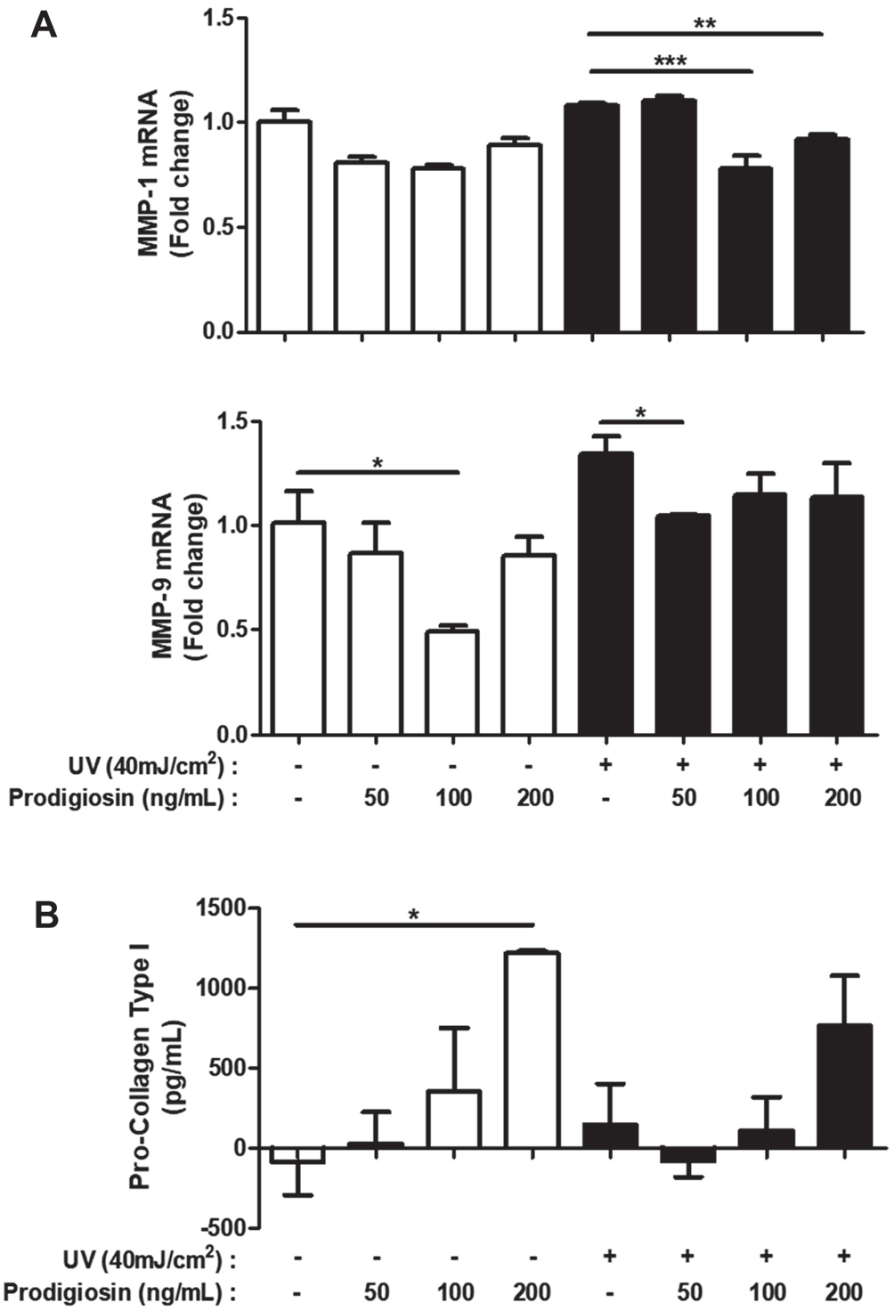
Prodigiosin inhibits photoaging in UV-irradiated HaCaT cells. HaCaT cells were irradiated with 40 mJ/cm^2^ UV light and treated with 0−200 ng/ml prodigiosin for 1 h. (**A**) mRNA levels of MMP-1 and MMP-9 were determined by RTqPCR in non-irradiated (white bars) and UV-irradiated (black bars) cells. (**B**) Levels of pro-collagen type I in culture media of non-irradiated (white bars) and UV-irradiated (black bars) cells were analyzed using a Human Pro-Collagen I alpha 1 ELISA Kit. Data represent mean ± SEM of two independent experiments. *p < 0.05, **p < 0.001, and ***p < 0.0001 determined by the one-tailed (**A**) or two-tailed (**B**) Student’s *t*-test.

UV radiation-induced ROS generation is a major cause of aging-related skin injury [18]. Synthesis and expression of matrix metalloproteinases (MMPs) are increased, and synthesis of collagen I is decreased [25], in photoaged skin [26, 27]. UV-irradiated (40 mJ/cm^2^) and non-irradiated HaCaT cells were treated with prodigiosin (0–200ng/ml) for 1 h, and mRNA expression of MMP-1 (collagenase) and MMP-9 (gelatinase) was investigated by RT-qPCR. Prodigiosin significantly decreased mRNA expression of MMP-1 and MMP-9 in UV-irradiated cells in a dose-dependent manner (Fig. 3A). Treatment with 100 ng/mL prodigiosin even markedly decreased mRNA expression of these two genes in non-irradiated cells (mRNA expression of MMP-1 and MMP-9 was decreased from 1-fold in non-treated cells to 0.7- and 0.5-fold in prodigiosin-treated cells, respectively)(Fig. 3A, white bars). Treatment with each concentration of prodigiosin reduced mRNA expression of MMP-1 and MMP-9 in UV-irradiated cells, such that it was similar to that in non-irradiated cells (Fig. 3A, black bars).

The level of pro-collagen type I protein was measured in the culture media of UV-irradiated and non-irradiated HaCaT cells treated with prodigiosin. Prodigiosin dose-dependently elevated the pro-collagen type I level in the culture media of UV-irradiated (black bars) and non-irradiated (white bars) cells (Fig. 3B). These results are consistent with the findings regarding mRNA expression of MMP-1 and MMP-9, and suggest that prodigiosin inhibits photoaging in UV-irradiated HaCaT cells. UV irradiation is the major cause of photoaging and changes the skin structure. We found that ROS production upon UV irradiation correlated with the levels of MMPs and pro-collagen type I, and that prodigiosin altered these levels to sustain the skin structure. These results provide evidence that prodigiosin may be useful to inhibit photoaging in keratinocytes.

### Prodigiosin Inhibits Inflammation in UV-Irradiated Keratinocytes

**Fig. 4:**
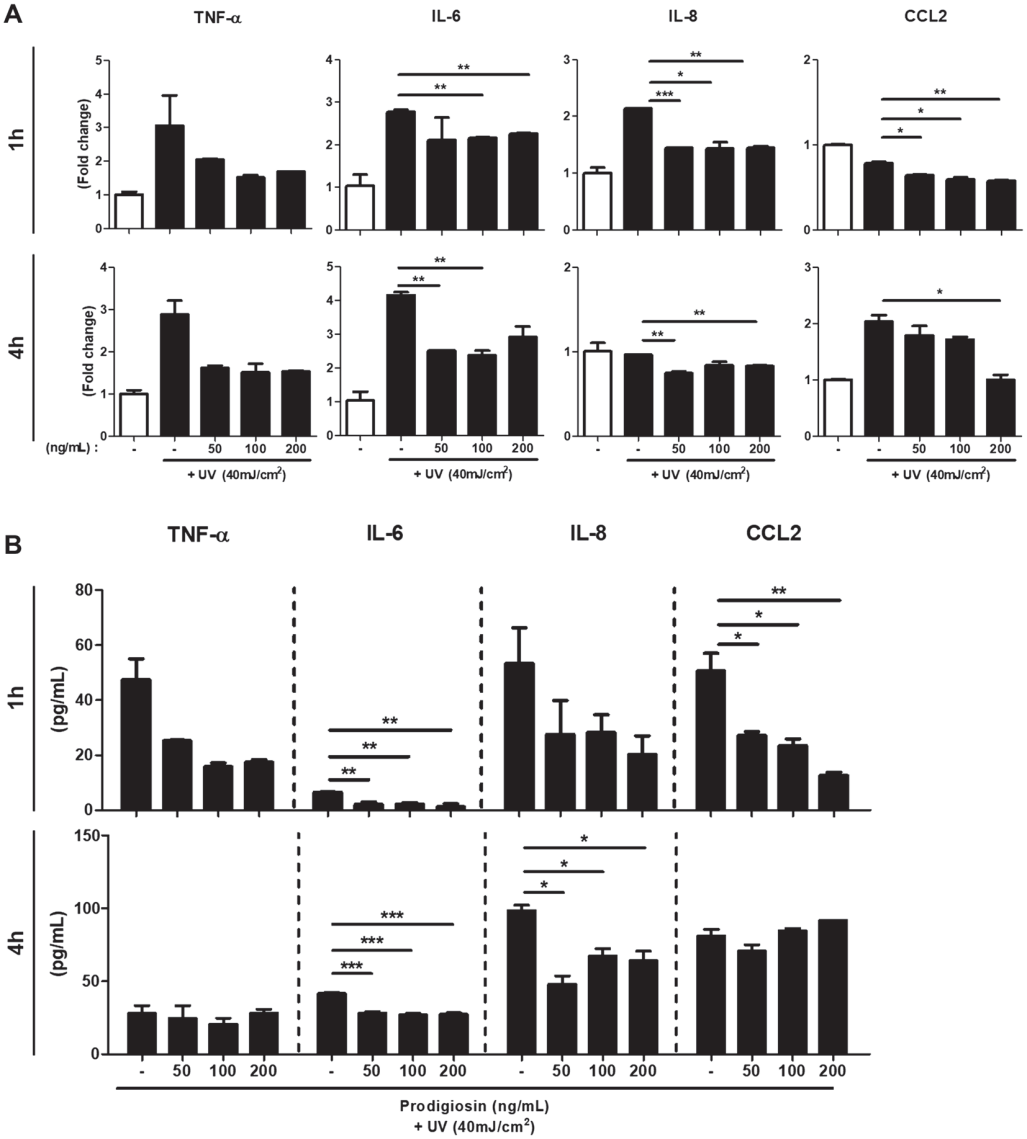
Prodigiosin reduces the inflammatory response in UV-irradiated HaCaT cells. HaCaT cells were irradiated with 40 mJ/cm^2^ UV light and treated with 0−200 ng/ml prodigiosin for 1 or 4 h. (**A**) mRNA levels of TNF-α, IL-6, IL- 8, and CCL2 were determined by RT-qPCR in non-irradiated (white bar) and UV-irradiated (black bars) cells. (**B**) Levels of TNF-α, IL-6, IL-8, and CCL2 proteins in culture media of cells were measured using ELISAs. Data represent mean ± SEM of three independent experiments. Statistical analyses were performed using the two-tailed Student’s *t*-test. *p < 0.05, **p < 0.001, and ***p < 0.0001.

Keratinocytes synthesize and secrete immunoregulatory proteins such as cytokines and chemokines in response to injury and external stimuli [28], including UV radiation. These proteins induce inflammation and regulate the immune response [29]. To further elucidate the activities of prodigiosin in UV-irradiated HaCaT cells, we studied its effects on the mRNA and protein levels of chemokines and proinflammatory cytokines. Cells were treated with 50, 100, and 200 ng/ml prodigiosin for 1 or 4 h after UV irradiation and lysed. mRNA levels of TNF-α, IL-6, IL-8, and CCL2 were determined by RT-qPCR. Although mRNA expression of the cytokine TNF-α was least affected by prodigiosin (Fig. 4A), it was still reduced to a similar level as that in non-irradiated control cells (Fig. 4A, white bars). Treatment with prodigiosin for 1 and 4 h significantly decreased mRNA expression of IL-6, IL-8, and CCL2 in UV-irradiated cells in a dose- and time-dependent manner (Fig. 4A).

Culture media were harvested and the concentrations of TNF-α, IL-6, IL-8, and CCL2 were measured by ELISAs. Prodigiosin dose- and time-dependently decreased expression of these proteins, which cause inflammation except for TNF-α and CCL2 (Fig. 4B), which is consistent with its effects on their mRNA levels determined by RT-qPCR (Fig. 4A). Treatment with 50 ng/ml prodigiosin for 1 h strongly reduced the protein levels of TNF-α, IL-6, IL-8, and CCL2. Several studies concluded that UV-irradiated human keratinocytes and keratinocyte cell lines release the proinflammatory cytokine IL-6 [30]. UV irradiation increased production of IL-6, and this was significantly inhibited by prodigiosin. These results suggest that prodigiosin is a useful cosmetic ingredient to suppress skin inflammation.

## Figures and Tables

**Table 1:** RT-qPCR primers

Gene (human)		Primers (5’-3’)
CCND2	Forward	TCATAGCAGCCACCTTCATTC
	Reverse	CTCTGCACTGAGATCTTCCTATTG
CDKN1A^*^	Forward	CAGCAGAGGAAGACCATGTG
	Reverse	GGCGTTTGGAGTGGTAGAAA
NRF2	Forward	GCAGACATTCCCGTTTGTAGA
	Reverse	AGGTGACTGAGCCTGATTAGTA
MMP-1	Forward	AGATGAAAGGTGGACCAACAA
	Reverse	GGTGTAGCTAGGGTACATCAAAG
MMP-9	Forward	CATGTACCCTATGTACCGCTTC
	Reverse	GTGTGGTGGTGGTTGGAG
TNF-α	Forward	CAGCCTCTTCTCCTTCCTGAT
	Reverse	GCCAGAGGGCTGATTAGAGA
IL-6	Forward	GATGAGTACAAAAGTCCTGATCCA
	Reverse	CTGCAGCCACTGGTTCTGT
IL-8	Forward	CTGCGCCAACACAGAAATTAT
	Reverse	AAACTTCTCCACAACCCTCTG
CCL2	Forward	TCATAGCAGCCACCTTCATTC
	Reverse	CTCTGCACTGAGATCTTCCTATTG
ACTIN	Forward	CACTCTTCCAGCCTTCCTTC
	Reverse	GTACAGGTCTTTGCGGATGT

*Primer sequences were obtained from [31].
